# Improving survival and narrowing age disparities in mantle cell lymphoma across therapeutic eras

**DOI:** 10.1038/s41408-026-01648-y

**Published:** 2026-10-13

**Authors:** Malak Munir, John L. Vaughn, Sravani Rimmalapudi, Narendranath Epperla

**Affiliations:** 1 Department of Medicine, Rochester General Hospital, Rochester, NY USA; 2 Division of Hematology & Oncology, Westchester Medical Center, Valhalla, NY USA; 3 Division of Hematology and Hematologic Malignancies, Huntsman Cancer Institute, University of Utah, Salt Lake City, UT USA

**Keywords:** Epidemiology, Haematological diseases

To the Editor,

Mantle cell lymphoma (MCL) is an aggressive subtype of B-cell non-Hodgkin lymphoma that predominantly affects older adults, with a median age at diagnosis of approximately 67 years and a marked male predominance [1, 2]. Treatment outcomes have improved substantially over the past two decades with the incorporation of rituximab, cytarabine-based induction and autologous stem cell transplantation (ASCT) in selected patients [3], and Bruton tyrosine kinase inhibitors (BTKis) [4]. More recently, CAR-T therapies [5] have expanded options for relapsed/refractory disease, while randomized studies have challenged the necessity of ASCT in selected patients [6, 7]. However, population-level survival gains across successive treatment eras, particularly among older, often transplant-ineligible patients, remain poorly defined, and the impact of therapeutic advances beyond the early BTKi era is unclear [8]. We therefore used the SEER-17 database to evaluate trends in MCL-specific mortality and survival from 2000 to 2023 across four therapeutic eras, with a focus on age-stratified outcomes.

Data were obtained from the SEER-17 database, covering approximately 26.5% of the US population. We included adults (≥ 18 years) with a first primary, pathologically confirmed MCL (ICD-O-3 code 9673/3) diagnosed between 2000 and 2023. Patients with central nervous system involvement (primary sites C70–C72) and those with missing survival data were excluded.

Patients were stratified into four groups based on eras of practice-changing therapies: 2000–2006 (rituximab integration, era-1), 2007–2013 (intensified immunochemotherapy, including bendamustine-rituximab adoption and ASCT consolidation, era-2), 2014–2019 (first-generation BTKi, era-3), and 2020–2023 (expanded targeted therapy and CAR-T, including zanubrutinib and brexucabtagene autoleucel, era-4). Patients were further classified by age at diagnosis, given the distinct treatment approaches across age groups, particularly ASCT eligibility. The study was conducted in compliance with the Declaration of Helsinki. Given the nature of the study (population-based study from a public repository), this study was IRB-exempt. As this was a retrospective study, informed consent was waived.

The primary outcomes were relative survival (RS), MCL-specific survival, and overall survival (OS). Cumulative incidence functions (CIF) for MCL-specific death and death from other causes were estimated with competing-risks methods and compared using Gray’s test. Adjusted subdistribution hazard ratios (SHR) were estimated using Fine-Gray regression, treating death from other causes as a competing event, with covariates including age (continuous), sex, race/ethnicity, stage, and era. Cox regression estimated hazard ratios for OS adjusted for the same covariates. To assess whether the era effect differed by age, we fitted an interaction term between age group (≤65 vs >65) and era, with joint significance assessed by the Wald test, and fitted age-stratified models separately. A secondary analysis repeated the CIF and regression models across four age subgroups (≤65, 66–74, 75–84, ≥ 85). RS was estimated using the Pohar-Perme method with US life tables matched by age, sex, race, and calendar year. Analyses were performed in R (version 4.5.3); tests were two-sided with *P* < 0.05 considered significant.

We included 12,785 patients with MCL diagnosed between 2000 and 2023 (Table 1). Mean age at diagnosis was 67.2 years (SD 11.6), 71.1% of patients were male, and 77.7% were non-Hispanic White. Patients aged ≤ 65 years comprised 44.0% of the cohort, while 56.0% were > 65 years. The distribution across eras was as follows: 2816 (22.0%) in era-1, 3657 (28.6%) in era-2, 3742 (29.3%) in era-3, and 2570 (20.1%) in era-4. The median follow-up was 3.3 years. During follow-up, 7578 patients died, including 5283 (69.7%) from MCL and 2295 (30.3%) from other causes.

Three-year RS improved progressively across treatment eras, increasing from 63.8% in era-1 to 68.7%, 71.9%, and 73.6% in eras 2–4, respectively. Concurrently, the 3-year cumulative incidence of MCL-specific death declined from 34.2% in era-1 to 29.9%, 24.7%, and 22.8% in eras 2–4, respectively (Gray’s *P* < 0.001; Fig. 1), corresponding to an absolute reduction of 11.4 percentage points and a relative reduction of 33.3%. In contrast, the 3-year cumulative incidence of death from other causes increased modestly from 7.5% in era-1 to 8.9% in era-4 (Gray’s *P* < 0.001).

MCL-specific mortality differed significantly by age. Across all eras, the 3-year CIF of MCL-specific death was lower among patients ≤ 65 years than those > 65 years (19.7% vs 35.4%; Gray’s *P* < 0.001). Mortality declined over time in both groups, from 24.2% to 14.8% (38.8% relative reduction) among patients ≤ 65 years and from 42.5% to 28.5% (32.9% relative reduction) among those > 65 years (Fig. S1). The age-by-era interaction was significant (*P* = 0.009), suggesting differential improvements across age groups.

In age-stratified analyses, the 3-year cumulative incidence of MCL-specific death declined across eras among all age groups (Table S1, Fig. S2). Among patients aged 66–74 years, mortality decreased from 33.1% in era-1 to 21.0% in era-4, while among those aged 75–84 years, it decreased from 47.7% to 31.9% (both Gray’s *P* < 0.001). Patients aged ≥ 85 years had the highest mortality throughout, with a more modest decline from 60.6% to 52.1% (Gray’s *P* = 0.038).

**Fig. 1:**
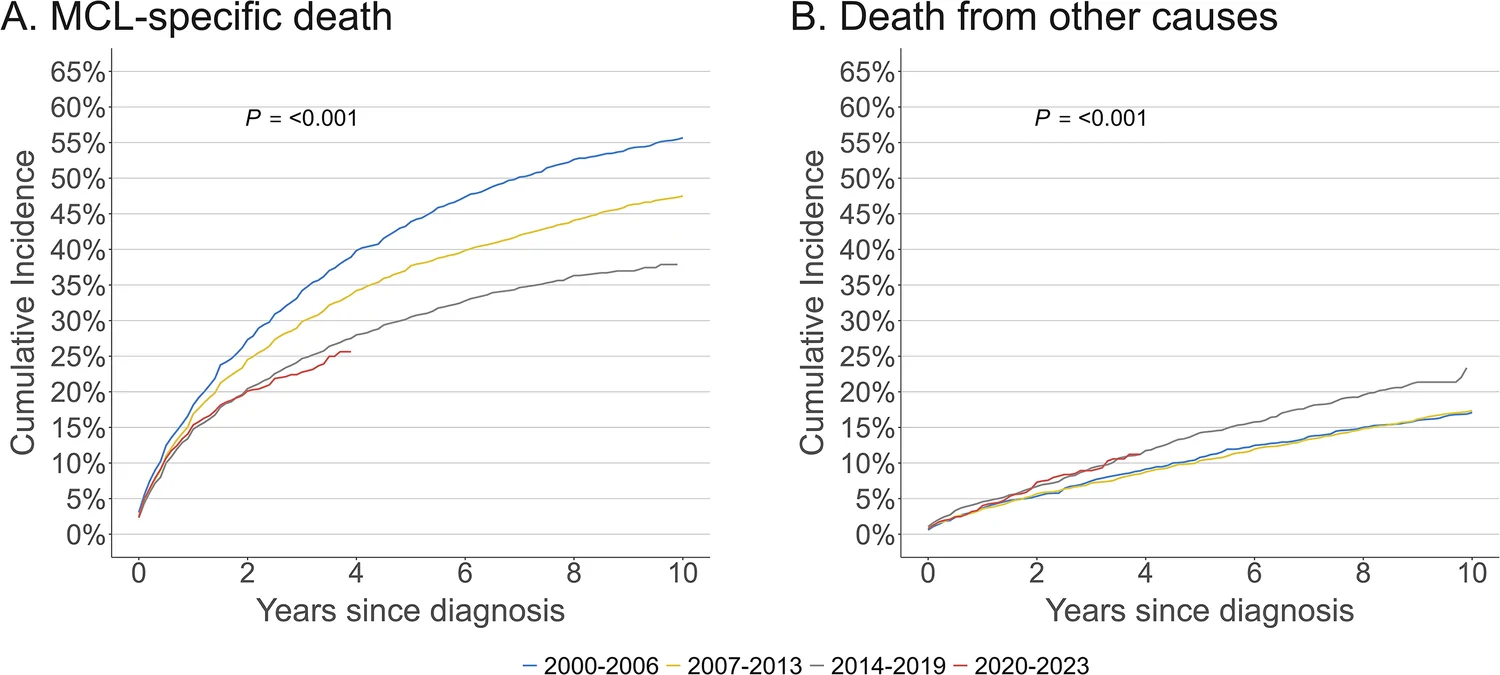
Cumulative incidence of MCL-specific death and death from other causes across therapeutic eras. Cumulative incidence functions estimated using the competing-risks method for (**A**) MCL-specific death and (**B**) death from other causes, stratified by era of diagnosis (2000–2006, 2007–2013, 2014–2019, 2020–2023).

**Table 1:** Baseline Characteristics of Study Cohort.

Characteristic	Total (N = 12785)^a^	2000–2006 (N = 2816)^a^	2007–2013 (N = 3657)^a^	2014–2019 (N = 3742)^a^	2020–2023 (N = 2570)^a^	*p*-value^b^
**Age, years**	67.2 (11.6)	66.5 (12.2)	66.8 (11.8)	67.7 (11.2)	68.0 (11.0)	< 0.001
**Sex**						0.12
Male	9096 (71.1%)	1972 (70.0%)	2580 (70.5%)	2673 (71.4%)	1871 (72.8%)	
Female	3689 (28.9%)	844 (30.0%)	1077 (29.5%)	1069 (28.6%)	699 (27.2%)	
**Race/Ethnicity**						< 0.001
Hispanic	1573 (12.3%)	252 (8.9%)	420 (11.5%)	484 (12.9%)	417 (16.2%)	
NHB	525 (4.1%)	108 (3.8%)	142 (3.9%)	154 (4.1%)	121 (4.7%)	
NHW	9933 (77.7%)	2321 (82.4%)	2901 (79.3%)	2853 (76.2%)	1858 (72.3%)	
Other	754 (5.9%)	135 (4.8%)	194 (5.3%)	251 (6.7%)	174 (6.8%)	
**Age Group Binary**						< 0.001
>65	7159 (56.0%)	1540 (54.7%)	1964 (53.7%)	2144 (57.3%)	1511 (58.8%)	
≤65	5626 (44.0%)	1276 (45.3%)	1693 (46.3%)	1598 (42.7%)	1059 (41.2%)	
**Age Group**						< 0.001
≤65	5,626 (44.0%)	1276 (45.3%)	1693 (46.3%)	1598 (42.7%)	1059 (41.2%)	
66–74	3498 (27.4%)	707 (25.1%)	932 (25.5%)	1092 (29.2%)	767 (29.8%)	
75–84	2768 (21.7%)	654 (23.2%)	766 (20.9%)	777 (20.8%)	571 (22.2%)	
85+	893 (7.0%)	179 (6.4%)	266 (7.3%)	275 (7.3%)	173 (6.7%)	
**Stage**						< 0.001
I	790 (6.2%)	146 (5.2%)	322 (8.8%)	173 (4.6%)	149 (5.8%)	
II	720 (5.6%)	114 (4.0%)	258 (7.1%)	176 (4.7%)	172 (6.7%)	
III	1538 (12.0%)	207 (7.4%)	520 (14.2%)	358 (9.6%)	453 (17.6%)	
IV	6446 (50.4%)	795 (28.2%)	2342 (64.0%)	1689 (45.1%)	1620 (63.0%)	
Unknown	3291 (25.7%)	1554 (55.2%)	215 (5.9%)	1346 (36.0%)	176 (6.8%)	
**Chemotherapy**^**c**^	9140 (71.5%)	2034 (72.2%)	2720 (74.4%)	2622 (70.1%)	1764 (68.6%)	< 0.001
**Radiation**^**c**^	899 (7.0%)	338 (12.0%)	272 (7.4%)	165 (4.4%)	124 (4.8%)	< 0.001

*NHB* non-Hispanic Blacks, *NHW* non-Hispanic Whites.

^a^Mean (SD); *n* (%).

^b^Kruskal-Wallis rank sum test; Pearson’s Chi-squared test.

^c^Chemotherapy and radiation reflect receipt of each modality as part of first-course treatment per SEER coding. The SEER chemotherapy variable does not reliably capture oral targeted agents (e.g., BTK inhibitors); rates in later eras therefore likely underestimate systemic therapy use.

In multivariable Fine-Gray models adjusted for age, sex, race, and stage, treatment era was independently associated with lower MCL-specific mortality (Table S2). Compared with era-1, SHRs were 0.77, 0.54, and 0.52 for eras 2–4, respectively (all *P* < 0.001). In age-stratified analyses, the reduction in MCL-specific mortality in era-4 versus era-1 was greatest among patients aged 66–74 years (SHR, 0.48; 95% CI, 0.39–0.59) and 75–84 years (SHR, 0.54; 95% CI, 0.44–0.66), and remained significant, albeit attenuated, among those aged ≥ 85 years (SHR, 0.68; 95% CI, 0.50–0.92). Notably, patients aged ≥85 years derived no significant benefit in era-2 (SHR, 1.04; *P* = 0.74), whereas mortality declined significantly in subsequent eras (Table S3). By era-4, the association between treatment era and MCL-specific mortality was similar in patients ≤ 65 and > 65 years (SHR, 0.53 for both; *P* < 0.01). OS demonstrated a consistent pattern, with adjusted hazard ratios of 0.83, 0.69, and 0.69 for eras 2–4, respectively, versus era-1 (all *P* < 0.001; Table S4).

Using SEER-17 data through 2023, we demonstrate a steady decline in MCL-specific mortality across successive therapeutic eras, providing population-level evidence that advances in MCL treatment have translated into meaningful survival gains. Although improvements were observed across all age groups, a significant age-by-era interaction (*P* = 0.009) suggested that the magnitude of benefit varied with age. The greatest reductions in MCL-specific mortality occurred among patients aged 66–74 and 75–84 years, while gains among those aged ≥85 years were comparatively attenuated, highlighting persistent challenges in the oldest patient population.

In a prior SEER analysis, only modest improvement in 5-year RS was noted between 2000–2006 and 2007–2013, with gains confined to patients aged 50–74 and no improvement among those aged ≥ 75 [9]. By extending follow-up through 2023 and applying a competing-risks framework, we found this apparent stagnation among older patients did not persist. Patients aged ≥ 85 experienced no significant improvement during 2007–2013, mirroring that analysis, but significant reductions in MCL-specific mortality emerged in the BTKi era (2014–2019) and were sustained in 2020–2023.

Similarly, Di et al. compared pre- and post-BTKi eras and reported reduced MCL-specific mortality among patients aged 60–79 but not those aged ≥ 80 [8]. By extending follow-up through 2023 and examining four distinct eras, we observed significant reductions even among patients aged ≥ 85 in both the BTKi and subsequent targeted/cellular therapy eras. These benefits were not apparent in earlier analyses [10–12], likely owing to their binary era comparisons, more limited follow-up, and lack of adjustment for the competing risk of non-MCL death; particularly relevant in an aging population where nearly one-third of deaths were from other causes.

The disproportionate era-3 survival gain among younger patients likely reflects broader access to cytarabine-based induction and ASCT consolidation, consistent with Castellino et al. [13]. By 2020–2023, however, this disparity narrowed substantially, with younger and older patients showing nearly identical reductions in MCL-specific mortality, coinciding with the expansion of effective, less age-restricted therapies including next-generation BTKis [14, 15] and brexucabtagene autoleucel [5]. While earlier trial data showed survival gains concentrated in younger patients [16], our findings suggest the age-related gap has begun to narrow at the population level. The modest rise in other-cause mortality across eras likely reflects improved MCL control and longer survival, with greater exposure to competing risks in an aging cohort.

In conclusion, this large population-based study of more than 12,000 patients with MCL demonstrates progressive and sustained improvements in MCL-specific survival across four distinct therapeutic eras spanning 2000–2023. These gains extend beyond younger populations and include meaningful improvements among older adults, particularly those aged 75–84 years, although benefits remain attenuated in patients aged ≥ 85 years. Importantly, the convergence of treatment-era effects between younger and older patients in the most recent era suggests that advances in targeted and cellular therapies may be beginning to mitigate longstanding age-related disparities in outcomes. Furthermore, the approval of non-covalent BTKi (pirtobrutinib) and BCL2 inhibitor (sonrotoclax), expanded use of CAR-T therapies, and promising activity of bispecific antibodies (glofitamab) will likely continue to reshape outcomes in MCL. Collectively, these findings indicate that the evolving therapeutic landscape is not only prolonging survival in MCL but may also be reshaping its age-dependent natural history at the population level.

## Supplementary information

Supplementary Appendix

## Data Availability

The data used in this study are publicly available through the Surveillance, Epidemiology, and End Results (SEER) Program of the National Cancer Institute (https://seer.cancer.gov).
