# Supplementary material for: Improving survival and narrowing age disparities in mantle cell lymphoma across therapeutic eras
Source: Blood Cancer J. 2026 Oct 13;16(1):182. doi: 10.1038/s41408-026-01648-y (PMC13661607; doi:10.1038/s41408-026-01648-y)
Supplement: Supplementary file 1 [file 41408_2026_1648_MOESM1_ESM.docx]

Contents

[Table S1. Cumulative Incidence of MCL-Specific Death by Era within Age Subgroups	2](#_Toc238824377)

[Table S2. Competing Risk Regression for MCL-Specific Death	4](#_Toc238824378)

[Table S3. Age-Subgroup Stratified Competing Risk Regression for MCL-Specific Death	5](#_Toc238824379)

[Table S4. Cox Proportional Hazards Regression for Overall Survival.	6](#_Toc238824380)

[Figure S1. Cumulative incidence of MCL-specific death by age group within each era.	7](#_Toc238824381)

[Figure S2. Age-stratified cumulative incidence of MCL-specific death.	8](#_Toc238824382)

# Table S1. Cumulative Incidence of MCL-Specific Death by Era within Age Subgroups

| **Age Group** | **Timepoint** | **Era** | **CIF (95% CI)** |
| --- | --- | --- | --- |
| **≤65** | **1-year** | 2000-2006 | 10.7% (9.9%-11.5%) |
|  |  | 2007-2013 | 9.8% (9.2%-10.4%) |
|  |  | 2014-2019 | 8.4% (7.9%-9.0%) |
|  |  | 2020-2023 | 9.6% (8.8%-10.5%) |
|  | **3-year** | 2000-2006 | 24.2% (22.6%-25.9%) |
|  |  | 2007-2013 | 21.4% (20.1%-22.7%) |
|  |  | 2014-2019 | 15.9% (14.9%-16.9%) |
|  |  | 2020-2023 | 14.8% (13.5%-16.3%) |
|  | **5-year** | 2000-2006 | 33.0% (30.8%-35.4%) |
|  |  | 2007-2013 | 27.8% (26.1%-29.5%) |
|  |  | 2014-2019 | 20.3% (19.1%-21.6%) |
| **66-74** | **1-year** | 2000-2006 | 17.3% (15.8%-18.9%) |
|  |  | 2007-2013 | 15.9% (14.6%-17.2%) |
|  |  | 2014-2019 | 14.2% (13.2%-15.3%) |
|  |  | 2020-2023 | 14.0% (12.7%-15.4%) |
|  | **3-year** | 2000-2006 | 33.1% (30.1%-36.4%) |
|  |  | 2007-2013 | 28.3% (26.1%-30.7%) |
|  |  | 2014-2019 | 24.8% (23.1%-26.8%) |
|  |  | 2020-2023 | 21.0% (18.7%-23.5%) |
|  | **5-year** | 2000-2006 | 46.8% (42.2%-51.9%) |
|  |  | 2007-2013 | 36.9% (33.9%-40.2%) |
|  |  | 2014-2019 | 32.0% (29.6%-34.6%) |
| **75-84** | **1-year** | 2000-2006 | 27.3% (24.8%-30.0%) |
|  |  | 2007-2013 | 24.4% (22.4%-26.7%) |
|  |  | 2014-2019 | 21.2% (19.4%-23.1%) |
|  |  | 2020-2023 | 22.8% (20.5%-25.5%) |
|  | **3-year** | 2000-2006 | 47.7% (42.8%-53.2%) |
|  |  | 2007-2013 | 41.1% (37.3%-45.2%) |
|  |  | 2014-2019 | 34.7% (31.6%-38.0%) |
|  |  | 2020-2023 | 31.9% (27.9%-36.4%) |
|  | **5-year** | 2000-2006 | 56.8% (50.5%-63.9%) |
|  |  | 2007-2013 | 51.3% (46.3%-56.9%) |
|  |  | 2014-2019 | 41.0% (37.3%-45.1%) |
| **85+** | **1-year** | 2000-2006 | 41.4% (34.0%-50.4%) |
|  |  | 2007-2013 | 44.0% (37.3%-51.9%) |
|  |  | 2014-2019 | 36.0% (30.8%-42.0%) |
|  |  | 2020-2023 | 32.3% (26.3%-39.7%) |
|  | **3-year** | 2000-2006 | 60.6% (47.8%-76.7%) |
|  |  | 2007-2013 | 56.8% (47.2%-68.3%) |
|  |  | 2014-2019 | 47.0% (39.8%-55.5%) |
|  |  | 2020-2023 | 52.1% (38.6%-70.4%) |
|  | **5-year** | 2000-2006 | 62.8% (49.3%-80.1%) |
|  |  | 2007-2013 | 64.3% (52.5%-78.7%) |
|  |  | 2014-2019 | 54.2% (45.3%-64.7%) |

Gray's test P-values: ≤65: <0.001; 66-74: <0.001; 75-84: <0.001; 85+: 0.038. 5-year estimates restricted to Eras 1-3 due to insufficient follow-up in 2020-2023.

# Table S2. Competing Risk Regression for MCL-Specific Death

| **Covariable** | **Unadjusted SHR (95% CI)** | **Adjusted SHR (95% CI)** | **P value** |
| --- | --- | --- | --- |
| Age (per year) |  | 1.04 (1.03-1.04) | <0.001 |
| Female (ref: Male) |  | 0.85 (0.80-0.90) | <0.001 |
| **Race/Ethnicity (ref: NHW)** |  |  |  |
| NHB |  | 1.11 (0.96-1.28) | 0.160 |
| Hispanic |  | 1.11 (1.02-1.21) | 0.019 |
| Other |  | 1.09 (0.96-1.22) | 0.180 |
| **Stage (ref: Stage I)** |  |  |  |
| II |  | 1.61 (1.35-1.92) | <0.001 |
| III |  | 2.00 (1.72-2.32) | <0.001 |
| IV |  | 2.03 (1.78-2.32) | <0.001 |
| Unknown |  | 1.91 (1.66-2.20) | <0.001 |
| **Era (ref: Era-1, 2000-2006)** |  |  |  |
| Era-2: 2007-2013 | 0.80 (0.75-0.85) | 0.77 (0.72-0.83) | <0.001 |
| Era-3: 2014-2019 | 0.59 (0.55-0.63) | 0.54 (0.50-0.58) | <0.001 |
| Era-4: 2020-2023 | 0.58 (0.52-0.64) | 0.52 (0.46-0.58) | <0.001 |

SHR = subdistribution hazard ratio; CI = confidence interval. Unadjusted models include era only. Adjusted model includes age (continuous), sex, race/ethnicity, stage, and era. Reference categories: Stage I, Era-1 (2000-2006), Male, Non-Hispanic White.

# Table S3. Age-Subgroup Stratified Competing Risk Regression for MCL-Specific Death

|  | **≤65**  **N = 5626** | | **66-74**  **N = 3498** | | **75-84**  **N = 2768** | | **85+**  **N = 893** | |
| --- | --- | --- | --- | --- | --- | --- | --- | --- |
| **Covariable** | **SHR (95% CI)** | **P value** | **SHR (95% CI)** | **P value** | **SHR (95% CI)** | **P value** | **SHR (95% CI)** | **P value** |
| Age (per year) | 1.03 (1.02-1.04) | <0.001 | 1.06 (1.04-1.09) | <0.001 | 1.03 (1.02-1.05) | <0.001 | 1.02 (0.97-1.06) | 0.500 |
| Female (ref: Male) | 0.80 (0.71-0.89) | <0.001 | 0.82 (0.73-0.92) | <0.001 | 0.91 (0.81-1.01) | 0.075 | 0.93 (0.78-1.10) | 0.390 |
| Race: NHB | 1.21 (0.98-1.48) | 0.076 | 1.08 (0.81-1.42) | 0.610 | 1.01 (0.73-1.40) | 0.970 | 0.99 (0.60-1.66) | 0.980 |
| Race: Hispanic | 1.19 (1.04-1.35) | 0.011 | 1.00 (0.85-1.19) | 0.960 | 1.07 (0.88-1.28) | 0.510 | 1.23 (0.91-1.66) | 0.180 |
| Race: Other | 0.97 (0.79-1.19) | 0.770 | 1.18 (0.95-1.48) | 0.130 | 1.29 (1.04-1.61) | 0.023 | 0.85 (0.58-1.24) | 0.400 |
| Stage: II | 1.38 (0.95-2.01) | 0.089 | 1.34 (0.97-1.85) | 0.081 | 1.84 (1.35-2.50) | <0.001 | 1.88 (1.28-2.75) | 0.001 |
| Stage: III | 1.83 (1.33-2.50) | <0.001 | 2.05 (1.57-2.69) | <0.001 | 2.10 (1.61-2.75) | <0.001 | 1.96 (1.40-2.74) | <0.001 |
| Stage: IV | 2.33 (1.76-3.07) | <0.001 | 2.12 (1.66-2.69) | <0.001 | 1.96 (1.54-2.51) | <0.001 | 1.50 (1.09-2.05) | 0.012 |
| Stage: Unknown | 2.24 (1.68-2.99) | <0.001 | 1.88 (1.46-2.42) | <0.001 | 1.76 (1.36-2.27) | <0.001 | 1.66 (1.19-2.31) | 0.003 |
| Era: 2007-2013 | 0.76 (0.67-0.86) | <0.001 | 0.71 (0.61-0.81) | <0.001 | 0.81 (0.71-0.93) | 0.003 | 1.04 (0.82-1.31) | 0.740 |
| Era: 2014-2019 | 0.48 (0.42-0.55) | <0.001 | 0.52 (0.46-0.60) | <0.001 | 0.60 (0.52-0.69) | <0.001 | 0.75 (0.59-0.95) | 0.016 |
| Era: 2020-2023 | 0.53 (0.43-0.65) | <0.001 | 0.48 (0.39-0.59) | <0.001 | 0.54 (0.44-0.66) | <0.001 | 0.68 (0.50-0.92) | 0.013 |

SHR = subdistribution hazard ratio; CI = confidence interval. Reference categories: Stage I, Era 2000-2006, Male, Non-Hispanic White. Models adjusted for age (continuous), sex, race/ethnicity, stage, and era.

# Table S4. Cox Proportional Hazards Regression for Overall Survival.

| **Covariable** | **HR (95% CI)** | **P value** |
| --- | --- | --- |
| Age (per year) | 1.06 (1.06-1.06) | <0.001 |
| Female (ref: Male) | 0.78 (0.74-0.82) | <0.001 |
| Race: NHB | 1.23 (1.10-1.38) | <0.001 |
| Race: Hispanic | 1.17 (1.09-1.26) | <0.001 |
| Race: Other | 1.01 (0.91-1.12) | 0.841 |
| Stage: II | 1.39 (1.21-1.60) | <0.001 |
| Stage: III | 1.68 (1.49-1.89) | <0.001 |
| Stage: IV | 1.69 (1.52-1.87) | <0.001 |
| Stage: Unknown | 1.69 (1.51-1.88) | <0.001 |
| Era: 2007-2013 | 0.83 (0.78-0.88) | <0.001 |
| Era: 2014-2019 | 0.69 (0.65-0.74) | <0.001 |
| Era: 2020-2023 | 0.69 (0.63-0.76) | <0.001 |

HR = hazard ratio; CI = confidence interval. Reference categories: Stage I, Era 2000-2006, Male, Non-Hispanic White.

Figure S1. Cumulative incidence of MCL-specific death by age group within each era. Competing-risks cumulative incidence functions for MCL-specific death, comparing patients ≤65 versus >65 years within each era of diagnosis: (A) 2000–2006, (B) 2007–2013, (C) 2014–2019, (D) 2020–2023.

Figure S2. Age-stratified cumulative incidence of MCL-specific death. Cumulative incidence functions for MCL-specific death estimated using the competing-risks method. (A–D) Incidence by age subgroup (≤65, 66–74, 75–84, ≥85 years) within each era of diagnosis. (E–H) Incidence by era of diagnosis within each age subgroup.
